# Re-Audit of The Anticholinergic Burden on A later Life Psychiatry Inpatient Ward

**DOI:** 10.1192/bjo.2024.643

**Published:** 2024-08-01

**Authors:** Cara Webb, Japsimar Kaur

**Affiliations:** Greater Manchester Mental Health Trust, Wythenshawe, United Kingdom

## Abstract

**Aims:**

To reassess and compare whether changes to medications are being made based on the anticholinergic effect on cognition (AEC) score. To understand the impact and implementation of recommendations from the first audit and whether these have led to change.

**Methods:**

Data on AEC scores were collected from one later life ward in Greater Manchester. The audit considered patients admitted September 2022 to January 2023 and the re-audit those admitted March 2023 to September 2023. The data was collected retrospectively by the auditors by going into the electronic notes of the initial ward round, the four week ward round and the patients' electronic prescription charts, information was then inputted into and analysed in Excel.

**Results:**

21 patients were included in the audit and 23 in the re-audit. Roughly 50% of patients in the initial audit scored 0 at 4 weeks, only 13% re-audit patients scored a 0. Eight more patients saw an increase in their AEC score within the re-audit than the audit. 5 patients had a lower anticholinergic burden during the audit, only 3 patients saw this decrease during the re-audit.

**Conclusion:**

Unfortunately it appears the first audit's recommendations were not adequately implemented as no improvement was seen between audits. We must therefore try to increase awareness of the adverse effects of anticholinergic medications through posters, teaching sessions by pharmacists and information posters on the ward; the effectiveness of these interventions can be analysed through a future quality improvement project.

The second recommendation is that the AEC score is to be calculated routinely for all patients admitted to an old age ward within the GMMH trust. This information to be included in the junior doctor induction pack and as a subcategory in the ward round documentation proforma.

A final recommendation is for the electronic prescribing system to include a built-in AEC calculator and prompt at admission and 4 weeks with the AEC score.

